# Performance Evaluation of a Piezoelectric Energy Harvester Based on Flag-Flutter

**DOI:** 10.3390/mi11100933

**Published:** 2020-10-14

**Authors:** Hassan Elahi, Marco Eugeni, Federico Fune, Luca Lampani, Franco Mastroddi, Giovanni Paolo Romano, Paolo Gaudenzi

**Affiliations:** Department of Mechanical and Aerospace Engineering (DIMA), Sapienza University of Rome, Via Eudossiana 18, 00184 Rome, Italy; marco.eugeni@uniroma1.it (M.E.); fune.1611352@studenti.uniroma1.it (F.F.); luca.lampani@uniroma1.it (L.L.); franco.mastroddi@uniroma1.it (F.M.); giampaolo.romano@uniroma1.it (G.P.R.); paolo.gaudenzi@uniroma1.it (P.G.)

**Keywords:** energy harvesting, piezoelectric material, flag-flutter, aeroelastic, fluid-structure interaction, smart structures, flow induced structural vibrations, flutter velocity

## Abstract

In the last few decades, piezoelectric (PZT) materials have played a vital role in the aerospace industry because of their energy harvesting capability. PZT energy harvesters (PEH) absorb the energy from an operational environment and can transform it into useful energy to drive nano/micro-electronic components. In this research work, a PEH based on the flag-flutter mechanism is presented. This mechanism is based on fluid-structure interaction (FSI). The flag is subjected to the axial airflow in the subsonic wind tunnel. The performance evaluation of the harvester and aeroelastic analysis is investigated numerically and experimentally. A novel solution is presented to extract energy from Limit Cycle Oscillations (LCOs) phenomenon by means of PZT transduction. The PZT patch absorbs the flow-induced structural vibrations and transforms it into electrical energy. Furthermore, the optimal resistance and length of the flag is predicted to maximize the energy harvesting. Different configurations of flag i.e., with Aluminium (Al) patch and PZT patch for flutter mode vibration mode are studied numerically and experimentally. The bifurcation diagram is constructed for the experimental campaign for the flutter instability of a cantilevered flag in subsonic wind-tunnel. Moreover, the flutter boundary conditions are analysed for reduced critical velocity and frequency. The designed PZT energy harvester via flag-flutter mechanism is suitable for energy harvesting in aerospace engineering applications to drive wireless sensors. The maximum output power that can be generated from the designed harvester is 6.72 mW and the optimal resistance is predicted to be 0.33 MΩ.

## 1. Introduction

The usage of self-powered electronics is increasing because of robustness in the design [1]. The advancement in technology specifically in Internet of Things (IoT) applications has resulted in a tremendous need for self-powered wireless sensors. As batteries are heavy and are expensive to maintain, and limited in capacity and life, there is therefore a need for mechanisms that can power the nano or microelectronics by absorbing structural energy [2]. The field of energy harvesting is of great interest for researchers with applications in aerospace industry [3]. There are many mechanisms for energy transformation i.e., electromagnetic [4], electromechanical [5,6], and fluid-structure interaction systems [7]. Among these mechanisms, fluid-structure interaction (FSI) systems play a vital role because of its voltage-dependent actuation [8,9,10]. Many researchers are working on such techniques to drive electronic circuits in electromechanical systems [11,12]. The piezoelectric material is mostly used to harvest electrical energy by absorbing mechanical energy (mechanical vibrations) from the surrounding [13,14].

The flapping flag instability in an inviscid steady axial flow is a classical problem of FSI [15]. Presently, the comprehension of this phenomenon is important for many applications such as paper processing [16], snoring [17], energy harvesting [18] and turbulence reduction [19]. In nature, many applications of flapping flag can be observed i.e., swimming of the fish [20]. In recent decades, this interaction mechanism between oscillating structures and fluid flows has become of particular importance [21]. At first, the flag-flutter phenomenon has been addressed by Rayleigh [15] from a theoretical point of view. He suggested that an elastic plate of infinite dimensions is always unstable when immersed in the axial potential flow. More recently Argentina et al. [22] studied the response of cantilevered plate in axial flow by considering the linear beam model based on classical Theodorsen’s theory. This model was designed to analyze the effect of boundary conditions as well as the investigation of the flapping plate sound prediction [23]. Alben et al. [24] presented a linearized model for this interaction phenomenon by considering a vortex sheet coupled to a flag of finite-length. This model represented that by reducing the rigidity of the plate, the high spatial-frequency modes tends to become unstable, leading to the increase in complexity of the non-linear dynamics. A full non-linear model was presented by Michelin et al. [25], in which the continuous vortex sheet shed by the flexible flag is replaced by the shedding of discrete point vortices with unsteady strengths. Other numerical models include the viscosity of fluid such as using Immersed Boundary Method (IBM) [26] applied to the problem of flapping plates [27]. Masoud et al. [28] use another hybrid computational approach that integrates the Lattice Boltzmann Model (LBM) for the dynamics of incompressible viscous fluids and the Lattice Spring Model (LSM) for the mechanics of elastic solids.

From the experimental point of view, flag-flutter was first explored by Taneda et al. [29]. They studied flags made of various materials and shapes in a wind tunnel induced to axial flow and their results provided a base for future exploration in this field. Taneda’s work seems to be the only systematic study in this field until the experiments on an elastic filament flapping in a flowing soap film [30] and on paper sheets flapping in a breeze [16]. These experiments were used to elucidate other aspects of the phenomena such as the bi-stability of the flapping and stationary states, and characterization of the transition curve. Moreover, Watanabe et al. [16] coupled their structural model with a two-dimensional compressible Navier-Stokes solver. Tang et al. [31] used a nonlinear structural model with not-extendibility to study cantilevered plates in axial flow. They used a Vortex Lattice Model (VLM) to calculate the aerodynamic lift over the plate. Tang et al. [32] and Doare et al. [33] used a nonlinear equation of motion of the plate using the non-extendibility condition and an unsteady lumped vortex model to calculate the pressure difference across the plate and both the instability and the post-critical behavior of the system were studied. While these works remain as studies of the fundamentals of this natural phenomenon, fluid-induced flapping has found applications in energy harvesting in recent years. The energy harvested from the flutter of a plate in an axial flow by making use of piezoelectric materials was demonstrated by Doare et al. [33], who derived the equations for fully coupled linear dynamics of the fluid-solid and electrical systems.

For a flag-flutter-based energy harvesting, phenomenon of flutter plays a critical role [34]. Many researchers showed their interest towards PZT transduction-based FSI system [35,36]. The parametric distributed model from a cantilever beam for energy harvesting via electromechanical mechanism is presented by Elvin et al. [37]. The classical aeroelastic model for the small angle of attack is presented by Theodorsen’s, which is based on flutter [38], while Beran et al. presented the aeroelastic model for the large angle of attack by adding quasi-steady approximation [39]. Muturi et al. presented that the amplitude of LCOs remains steady for the nonlinear aeroelastic system if they are initially excited over the critical value [40]. Elahi et al. carried out a study to stress on the true prediction of aerodynamic model, as the evaluation of the performance of PZT aeroelastic energy harvester (PAEH) is dependent on it [41].

By using PZT material for energy harvesting based on flag-flutter, in-depth dynamic details of FSI-based flag-flutter are needed. The aeroelastic instability of a flag in a potential flow has been explored both numerically and experimentally in this paper. In particular, the study is conducted by using different numerical models with increasing complexity. The numerical model allows a first evaluation of how different parameters can influence the stability of the flag, namely, the unperturbed state. The nature of the observed bifurcation is studied experimentally in subsonic wind-tunnel. Finally, an experimental study is conducted and the results are compared with the numerical one. The main observed feature of the studied bifurcation process is a hysteresis phenomenon, which suggests the presence of a sub-critical bifurcation. The main contribution of the present paper is to present an analysis of a flag-flutter phenomenon both in terms of stability margin parameters (flutter flow speed, frequencies, and characteristic geometrical parameters) and critical aeroelastic mode shapes; via numerical and experimental campaign. The comparison of power generated by various PAEHs based on flutter mechanism is represented in Table 1.

This research work is the advancement of the work by Eugeni et al. [42]. The current manuscript explains all the results of the numerical and experimental campaign carried out. The scientific novel contribution of this manuscript is extensive experimentation validated with numerical simulations for flutter mode vibrations. In this research, energy harvesting results are discussed both numerically and experimentally at variable resistance ranging from 0.01 MΩ to 1 MΩ. The evolution of generated voltage is also represented for different resistances. Moreover, the optimal resistance is predicted experimentally for maximizing the energy harvesting phenomenon. In the experimental campaign, the bifurcation diagrams are observed with Photron FASTCAM AX200 (Tokyo, Japan) to understand the modification of the flutter-mode vibration with the flow velocity along the observed bifurcation LCOs diagrams experimentally evaluated (PZT and Al patch attached). This evaluation is critical for the harvester performance because the bending mode is the most important for the energy harvesting maximization. The designed PZT energy harvester via flag-flutter mechanism is suitable for energy harvesting in aerospace engineering applications i.e., in high altitude platforms, stratospheric probe balloons, and unmanned aerial vehicles.

The paper is organized as follows: in Section 2, the numerical model is elaborated; in Section 3, the experimental set-up is discussed; in Section 4, results are presented and are compared with the experimental and numerical predictions. Finally, in Section 5 concluding remarks are given.

## 2. Numerical Model

**Figure 1:**
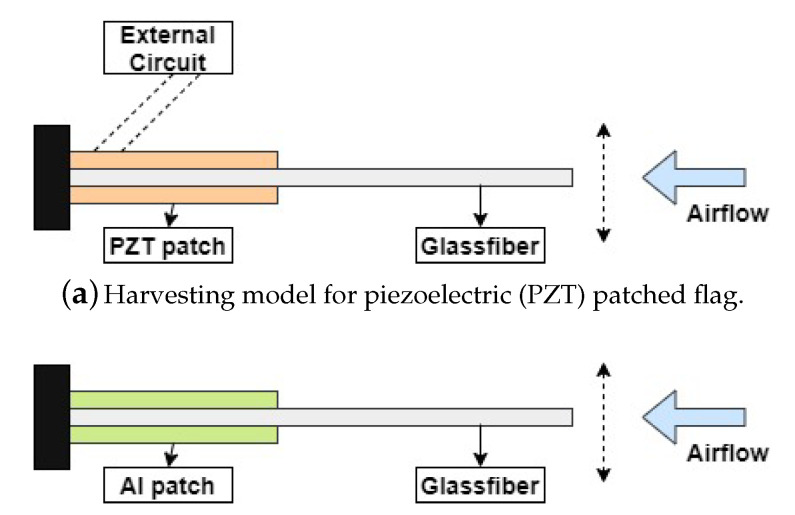
Overall energy harvesting model of flag-flutter subjected to axial flow.

In this paper, the PEH is based on the flag-flutter mechanism in which the harvester is considered to be a fiberglass plate as shown in Figure 1. For energy harvesting purposes, the bimorph configuration of PIC 255 (PZT material) is attached to the host structure provided with electronic circuits as shown in Figure 1a. For the validation of the designed energy harvesting model, another model is constructed. In this model, the same strategy is used as of PZT attached harvester except for two Aluminium (Al) patches without external circuits, which are attached to the fixed end of the host structure as shown in Figure 1b. These designed harvesters experience the airflow, resulting in LCOs, the PZT patches absorbs the flow-induced structural vibrations and transform them into useful electrical energy. The overall mechanism of PZT energy harvesting by the flag-flutter mechanism is shown in Figure 1. The geometry of the harvester is represented in Table 2 and the material properties of the harvester are shown in Table 3.

**Figure 2:**
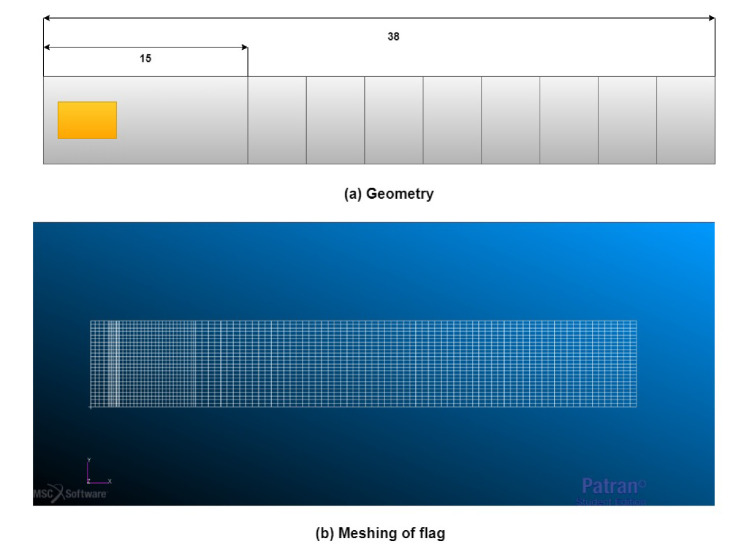
Numerical analysis of the flag-flutter mechanism based on flutter mechanism.

For numerical simulations, MSC Nastran software SOL 145 (2017, MSC Software, Newport Beach, CA, USA) is used for understanding the phenomenon of energy harvesting by the flag-flutter mechanism. The overall numerical model of the flag is represented in Figure 2. To have an in-depth knowledge of the harvesting mechanism, the modeled flags were of different lengths and configurations as represented in Figure 2a. The dimensions of the flag and PZT element is represented in Table 2. Three different configurations of the numerical model are constructed in this phase; (i) flag with Al patch attached, (ii) flag with no patch attached, and (iii) flag with PZT patch attached. These configurations are realized to predict the optimal output power and exploring of a wide range of airflow velocity for the flutter analysis.

The flag is considered to be cantilevered to duplicate the experimental conditions (carried out in subsonic wind tunnel). In particular, flags were discretized recurring to 2D rectangular planar bending elements. This was implemented in MSC Patran recurring to shell elements which are associated with CQUAD4s elements. Which defines isoparametric quadrilateral plate elements. Mesh was realised so that the displacement, in the connection and material changes area, could be well described with particular focus to the piezoelectric area. This required a thicker mesh in piezoelectric patch area, other parts of the flag were meshed in the way that the flutter motion is well described. Load conditions are adjusted in MSC Nastran input file, for this type of analysis the solver couples flow and structure, calculating for each value of the velocity interval.

The Doublet Lattice Method (DLM) is used for aeroelastic analysis. For the unsteady flow, the DLM is an extension of the steady vortex-lattice method (VLM) to unsteady flow. To analyze flow-induced structural vibrations, the solver couples the structure and flow, for every interval value of airflow velocity. The unknown lifting pressures are assumed to be concentrated uniformly across the one-quarter chord line of each aerodynamic panel as described in the modelling part as shown in Figure 2b. The total number of nodes and elements in the meshing of the flag during the numerical analysis is 1765 (17 cm) to 2625 (38 cm) and 1344 (17 cm) to 2496 (38 cm) respectively. The number of nodes and elements used in meshing during the numerical analysis are represented in Table 4.

For the energy harvesting mechanism, the prediction of the optimal length of the flag is a crucial element. Elahi et al. elaborated that for the working model of the PZT element, the voltage generated is directly proportional to the curvature of the flag [35]. It is evident from the research carried out by Eugeni et al. that a 29 cm flag is most optimized for energy harvesting mechanism via PZT transduction [42]. Therefore, a 29 cm length of the flag is selected for experiments.

## 3. Experimental Setup

**Figure 3:**
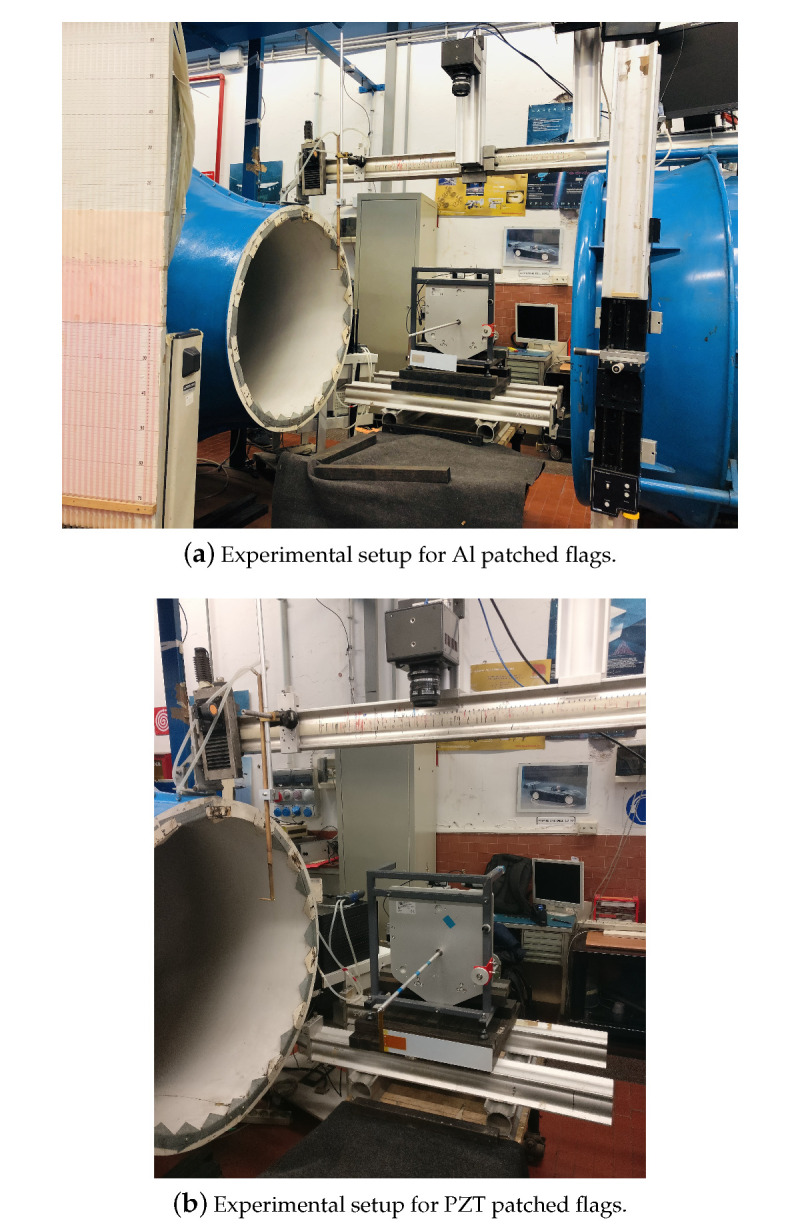
Overall experimental campaign for flag-flutter energy harvesting mechanism carried out in subsonic wind tunnel.

**Figure 4:**
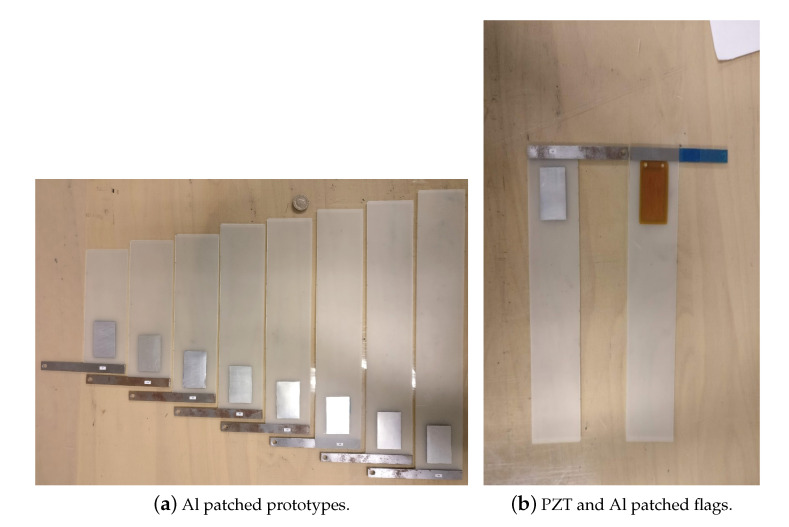
Al and PZT attached flags used for energy harvesting in subsonic wind tunnel.

**Figure 5:**
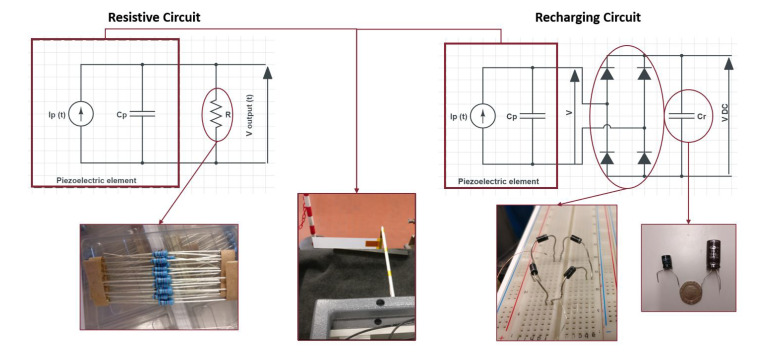
Overall circuit diagram for energy harvesting.

The experimentation of the current research is performed at Fluid and Dynamics Lab, Sapienza University of Rome, Italy. The subsonic wind-tunnel that has been used for the experimental campaign has a capacity of a maximum of 40 m/s airflow velocity, with a circular test cross-section of 0.9 m diameter. The pitot-static tube is used to control and measure the airflow velocity in a subsonic wind tunnel. To capture a flutter mode vibration of a harvester Photron FASTCAM AX200 (Tokyo, Japan) is used. It was mounted at the top of the subsonic wind-tunnel. The specifications of the camera are 5400 fps and 1024×736 pixel resolution. The images of the flutter mode vibration taken by the camera are used to predict the flutter amplitude. The harvester is subjected to the airflow which increases gradually from zero. For the small values of the airflow velocity, the plate was stable, until the critical flow velocity $U_{F}$ and the plate starts flapping. This flapping mode of the flag is recorded with a high-speed camera. The image processing technique was applied to these videos for the prediction of flapping frequency and amplitude. In this phase, flags with different configurations are used experimentally in subsonic wind-tunnel for the investigation of aeroelastic behavior, i.e, flag attached with PZT patch and flag attached with Al patch. The geometry of the harvester with the PZT and Al patch is represented in Table 5. The overall wind-tunnel experimental setup is represented in Figure 3. Al and PZT patched flags are shown in Figure 4. The overall circuit diagram of PZT energy harvesting via flag-flutter is represented in Figure 5.

## 4. Results and Discussion

In this research work, numerical and experimental campaign for the evaluation and prediction of the performance of flag-flutter-based PEH is carried out.

### 4.1. Energy Harvesting

In this phase, for the energy harvesting performance evaluation of a flag, the output voltage and power are measured both numerically as well as experimentally. The numerical model was designed in accordance with the work done by Elahi et al. [41]. For the aeroelastic energy harvesting solution, the motion of flag tip which is a driving factor for energy harvesting is expressed in Equation (1).

(1) $$q-k_{ME}\xi +C_{P}V=0$$

where the electrical charge on electrodes is represented by *q*, the electromechanical coupling factor is represented by $k_{ME}$, the flag tip displacement is represented by ξ, the PZT inherent capacitance is represented by $C_{p}$ and the electric potential between two electrodes is represented by *V*. The electromechanical coupling factor is defined in Equation (2).

(2) $$k_{ME}=\frac{dhe_{31}}{2}$$

where *d* is the PZT patch width, *h* is the flag thickness and the PZT constant in 31 coupling direction is represented by $e_{31}$. In this work, external resistance is considered parallel to the internal one, so:

(3) $$-R\dot{q}=v$$

where *R* is the external resistance in the circuit. Considering the sinusoidal tip motion, with an amplitude of *A*, as expressed in Equation (4).

(4) ξ=Asin(ωt)

Therefore, the differential problem and its solution are represented in Equations (5)–(8).

(5) $$\dot{q}=\frac{k_{ME}Asin\left(\omega t\right)-q}{RC_{p}}$$

(6) $$q=\frac{Ak_{ME}\left[\sin\left(\omega t\right)-RC_{p}\omega \cos\left(\omega t\right)+RC_{p}\omega \exp\left(-\frac{t}{RC_{p}}\right)\right]}{1+{\left(RC_{p}\omega \right)}^{2}}$$

(7) $$I_{p}=\dot{q}=\frac{Ak_{ME}\omega \left[\cos\left(\omega t\right)+RC_{p}\omega \sin\left(\omega t\right)-\exp\left(-\frac{t}{RC_{p}}\right)\right]}{1+{\left(RC_{p}\omega \right)}^{2}}$$

(8) $$V_{out}=\frac{Ak_{ME}R\omega \left[\cos\left(\omega t\right)+RC_{p}\omega \sin\left(\omega t\right)-\exp\left(-\frac{t}{RC_{p}}\right)\right]}{\sqrt{1+(RC_{p}\omega )}\left[1+{\left(RC_{p}\omega \right)}^{2}\right]}$$

The output power *P* is calculated then by using Ohm’s law as expressed in Equation (9). For experimental campaign, the circuit is represented in Figure 5.

(9) $$P=IV=\frac{V_{out}^{2}}{R}$$

**Figure 6:**
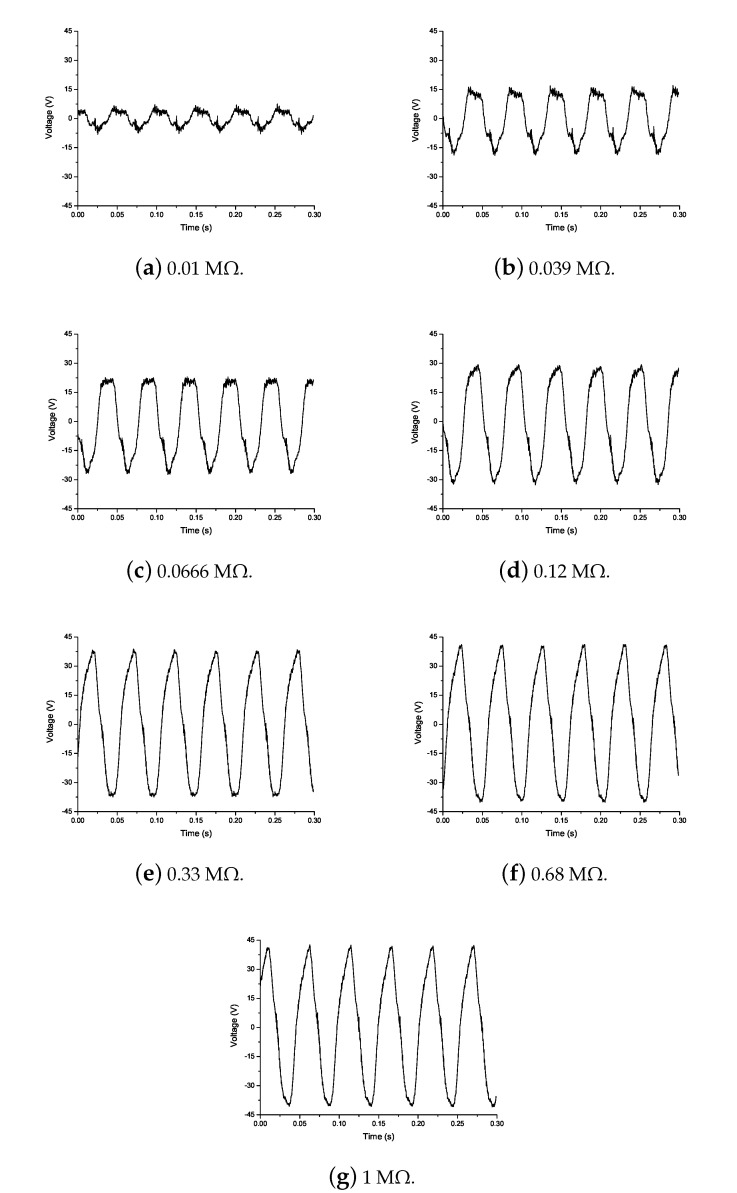
Experimental output voltage for different resistances.

The flag attached to the PZT patch was subjected to the airflow until the flutter velocity and phenomenon of LCOs occurs. The PZT patch absorbs flow-induced structural vibrations and transforms it into electrical energy. The voltage generated is measured by a cathode-ray oscilloscope. The experimental campaign is carried out from 0.01 MΩ to 1 MΩ external resistance, for the prediction of the optimal resistance, as represented in Figure 6. The increase in the amplitude of voltage generated can be observed from 0.01 MΩ to 0.33 MΩ resistance, after this point, the output voltage remains approximately constant. Thus, 0.33 MΩ resistance is considered to be an optimal resistance with a 35 V output voltage. The time response obtained experimentally represented a nonlinear behavior because the piezoelectric patch attached to the flag absorbed structural vibrations from limit cycle oscillations that are nonlinear in behavior, resulting in nonlinear voltage generation with respect to time.

**Figure 7:**
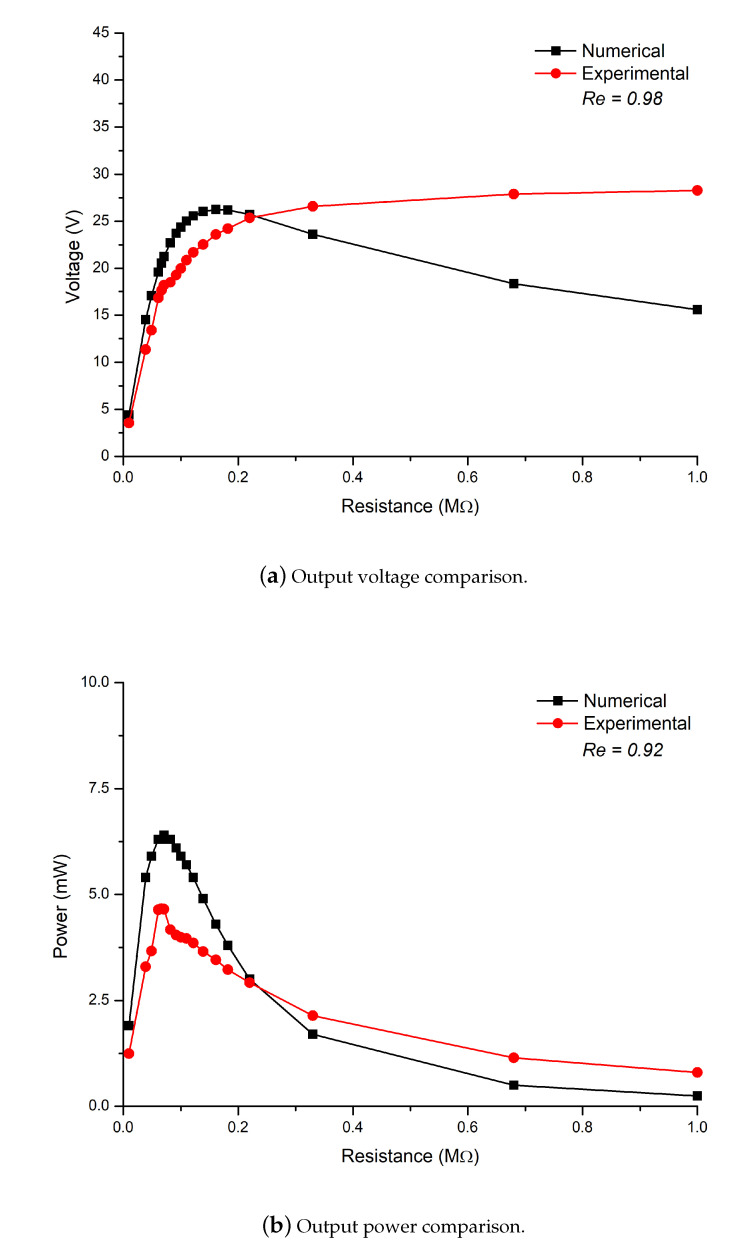
Output voltage and power generated comparison of numerical and experimental data with correlation value; where Re represents the correlation value.

To validate the experimental results with the numerical ones, a comparison has been carried out of numerical and experimental data for energy harvesting of the flag as shown in Figure 7. In Figure 7a, comparison of voltage generated by the PZT patch experimentally and numerically is carried out. The results show approximately the same trend for numerical and experimental data with a correlation value of 0.98. For low values of resistance, the experimental trends are well described by the semi-analytical one degree of freedom spring-mass damper model with no piezo-to-structure interaction. In Figure 7b, comparison of the output power of the PZT patch experimentally and numerically is carried out. The results show approximately the same trend for numerical and experimental data with a correlation value of 0.92. For low values of electrical resistance, the experimental trends are well described by the semi-analytical 1 degree of freedom spring-mass-damper model (no piezo-to-structure interaction). The numerical and experimental results show the same trend and the correlation was found to be 0.98 for voltage generation and 0.92 for power generation.

### 4.2. Flutter Vibration Mode

**Figure 8:**
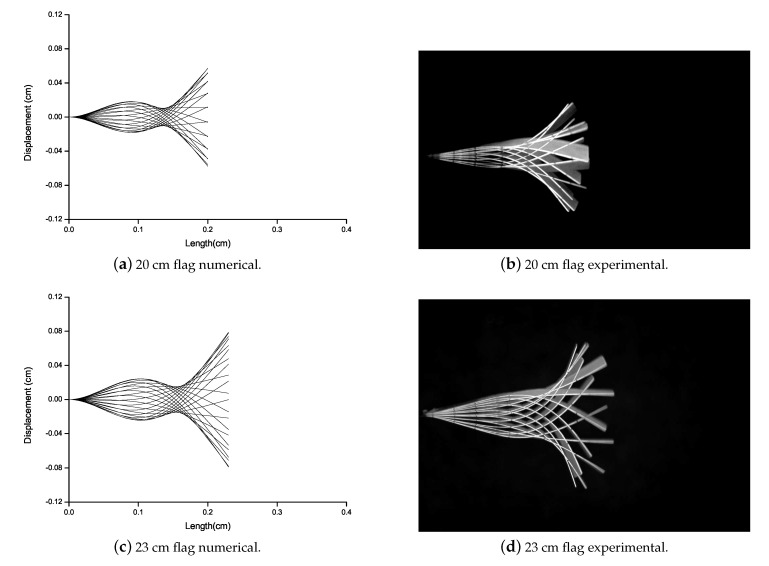

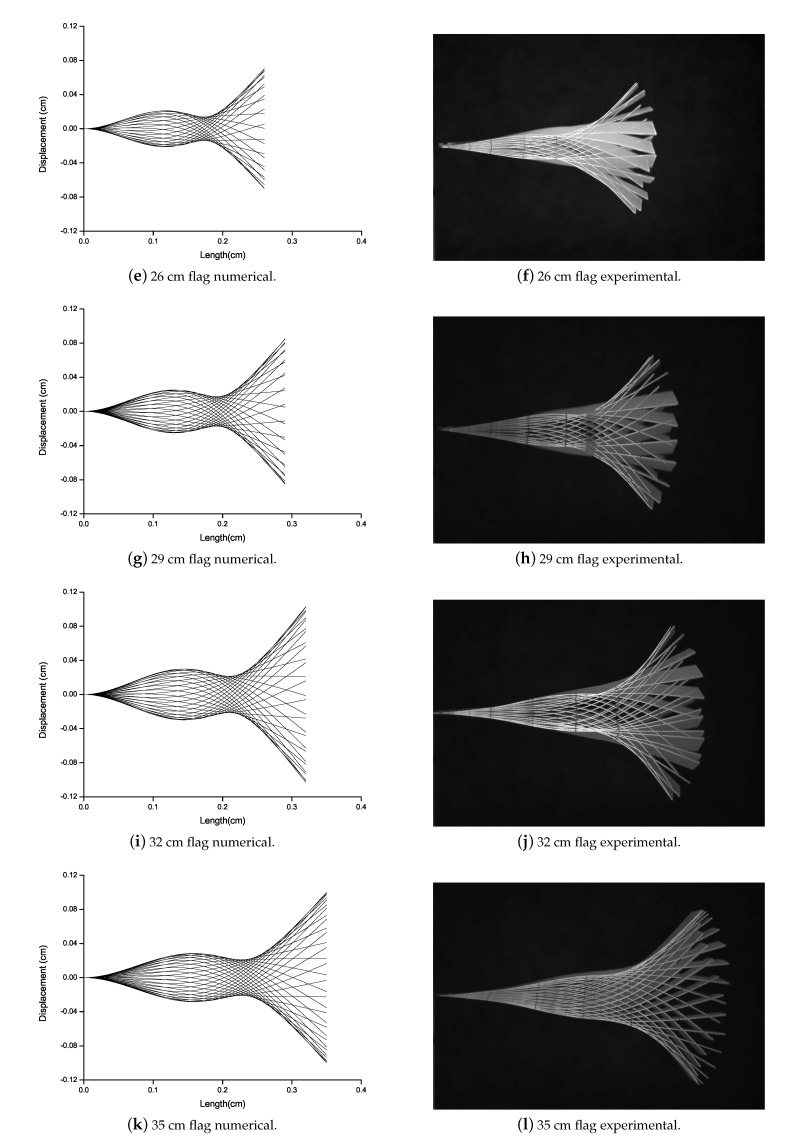

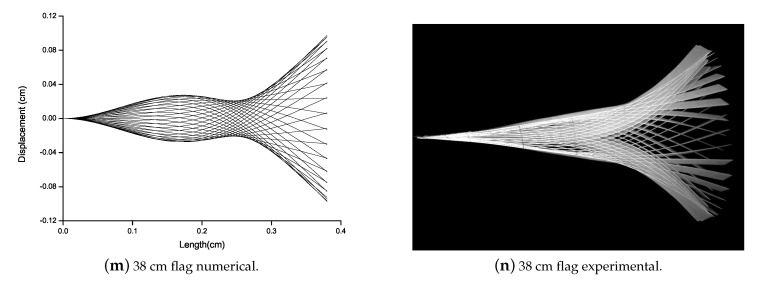
Deformation comparison for Al patched flags varying from 15 cm to 38 cm long flags.

Eugeni et al. [42] studied that when the flag is subjected to airflow, flutter occurs abruptly resulting in the flapping of the flag. In this work, to capture this flutter mode vibrations, numerical and experimental study is carried out. Flutter vibration mode of all the flags attached with the Al patch is analyzed numerically and experimentally as shown in Figure 8. For all the flags attached with the Al patch, there is no mode shape variation. The numerical and experimental data are good in agreement. As the torsion is not predicted numerically and with the larger length of the flag the torsion is more. Therefore, a difference can be observed between numerical and experimental results of 35 cm and 38 cm length of the flag.

**Figure 9:**
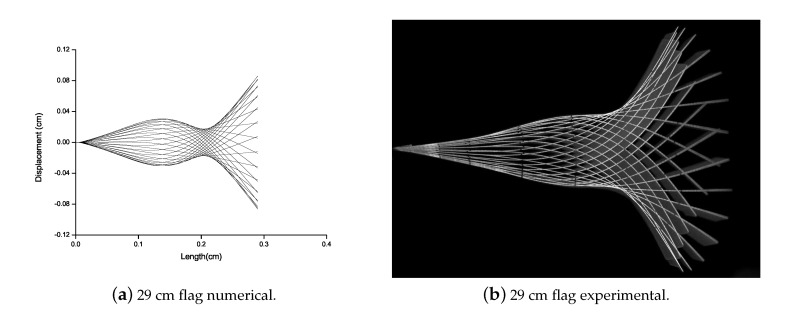
Deformation comparison for PZT patched flag.

Flutter vibration mode of the PZT patched flag is analyzed via numerical and experimental campaign as shown in Figure 9. There is no mode shape variation for the flag attached to the PZT patch. Therefore, the numerical and experimental results are good in agreement.

### 4.3. Bifurcation Analysis

To analyze the bifurcation diagram of the different configurations of the flag, an experimental campaign is carried out in this phase. In the literature, the bifurcation diagrams for the flags are studied by Eugeni et al. [42]. In order to have a better understanding of the flapping of the flag and to have an idea about the tip deflection of the flag, these bifurcation diagrams are observed with FASTCAM to understand the effect of the phenomenon of flutter velocity and LCOs on the flag (PZT and Al patch attached) in pictorial form, during the experimental campaign in the subsonic wind tunnel.

A strategy is developed for the construction of bifurcation diagram composing of following steps; (i) The flag is subjected to the airflow which increases gradually from zero until the flag is stable; (ii) by increasing the velocity there comes a point where flutter arises abruptly, flag starts flapping; (iii) by gradually increasing the velocity above the flutter velocity; (iv) by gradually decreasing the velocity below the flutter velocity until the flag becomes stable. This strategy is applied to the three configurations of the flag i.e., flag attached with Al patch, flag attached with PZT patch (optimal resistance), and flag attached with PZT patch (open circuit).

**Figure 10:**
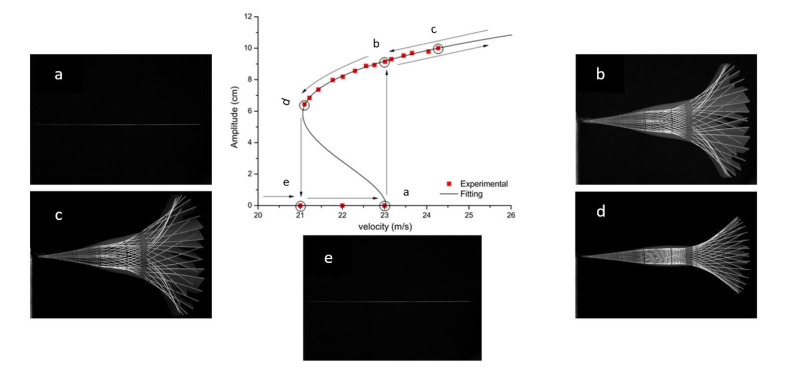
Bifurcation diagram for Al patched flag.

For the flag attached with the Al patch, a hysteresis loop is observed for the bifurcation diagram as shown in Figure 10. When the Al patched flag is subjected to airflow, it remains stable until 23 m/s as shown in Figure 10a. At this point, flutter arises abruptly and the flag starts flapping as shown in Figure 10b. When gradually increasing the velocity above the flutter velocity the amplitude of the vibrations increases as shown in Figure 10c. By gradually decreasing the velocity to the flutter velocity, it is observed that the flag still flaps but with a less amplitude as shown in Figure 10d. When the velocity is further decreased, the flag tends to stable again as shown in Figure 10e. In Figure 10, the hysteresis is observed representing a fifth-order fitting sub-critical bifurcation for the Al patched flag.

**Figure 11:**
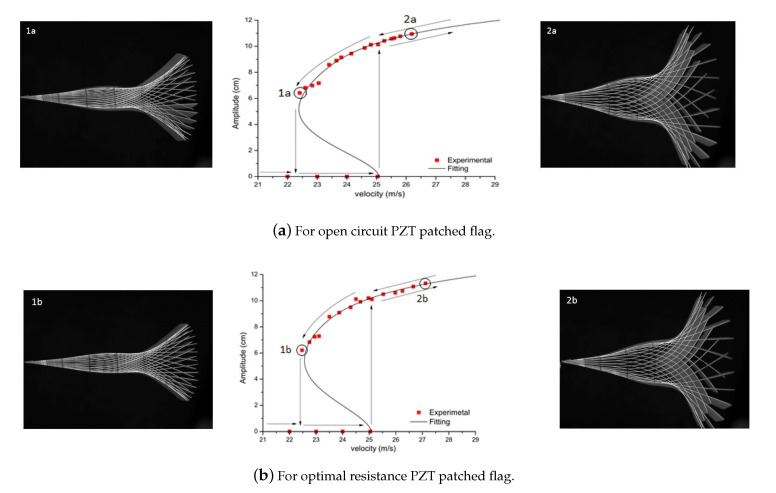
Bifurcation diagram for PZT patched flag.

The same strategy is applied to flag attached with PZT patches i.e., with open circuit (as shown in Figure 11a) and with optimal resistance (as shown in Figure 11b). In both cases, the flag is stable until 25 m/s, and at this point, flutter starts abruptly resulting in the flapping of flag. PZT patched flag for both cases represents the hysteresis loop. In Figure 11, the hysteresis is observed representing a fifth-order fitting sub-critical bifurcation for the PZT patched flag. The jump can be observed in both cases of bifurcation diagram as represented in Figure 11. It is suggested that this jump is due to the presence of an unstable branch or due to the effect of PZT interaction on the host structure.

### 4.4. Flutter Boundary Conditions

**Figure 12:**
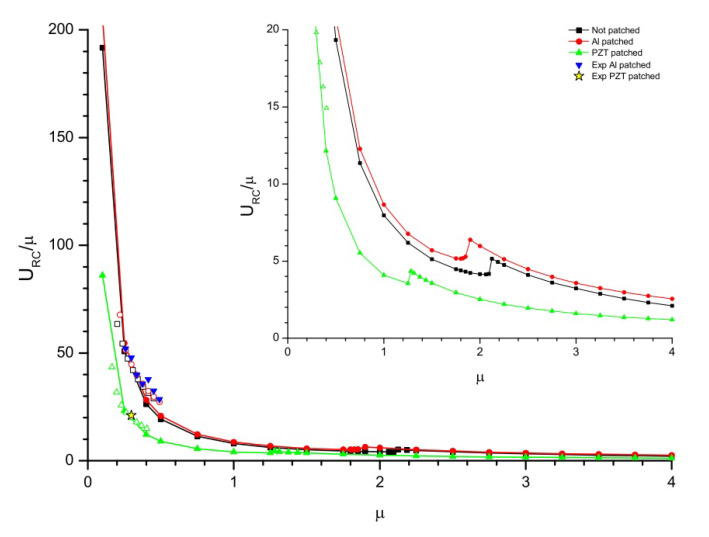
Flutter for reduced critical velocity; where $\mu =\rho_{F}L/\rho_{S}t$ and $U_{RC}/\mu =\left[{\left(\rho_{P}h\right)}^{3/2}/\left(\rho_{F}D^{1/2}\right)\right]U$.

In this phase, the flutter boundary conditions are analyzed for three configurations of flag i.e., flag with no patch attached, flag with Al patch attached, and flag with PZT patch attached as shown in Figure 12 are studied numerically and experimentally. This strategy was developed from the work of Tang et al. [32] to explore the flutter boundary conditions of the flag by altering the length of the flag as it is the most controllable parameter in experimentation. The numerical study is in good agreement with the experimental one. The flutter boundary conditions are expressed in terms of the mass ratio represented as μ, depending on the length of the flag. The mass ratio can be defined mathematically as $\mu =\rho_{F}L/\rho_{S}t$ where $U_{RC}/\mu =\left[{\left(\rho_{P}h\right)}^{3/2}/\left(\rho_{F}D^{1/2}\right)\right]U$ is an ordinate. Where $\rho_{F}$ and $\rho_{S}$ represents the density of airflow and flag respectively. From Figure 12 the relation between numerical and experimental data of the most controllable parameters (i.e., length of the flag and reduced critical velocity) are in good agreement. From the numerical and experimental data represented in Figure 12, it is observed that by increasing the flag length the flutter velocity reduces. However, within the range of 1.2:μ:1.4, 1.8:μ:2.0, and 2.0:μ:2.2 for flag attached with PZT patch, Al patch and no patch respectively, there is a local rise and then subsidence in $U_{RC}$ as *L* increases; a subtle transition in the flutter mode shape occurs in this interval.

**Figure 13:**
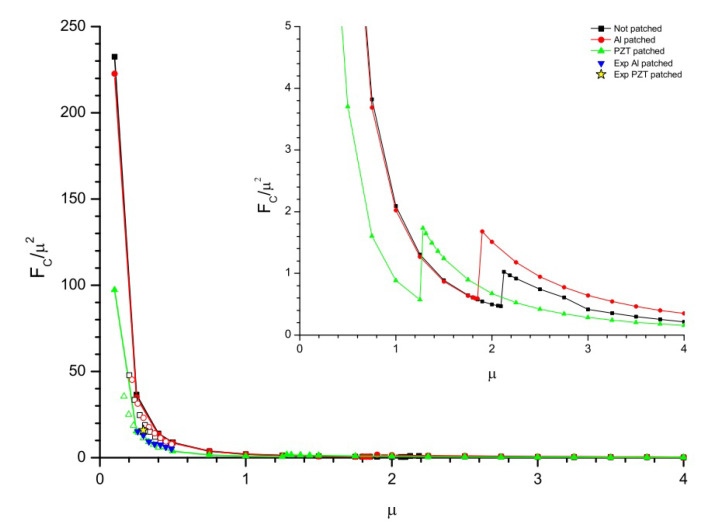
Flutter for reduced critical frequency; where $\mu =\rho_{F}L/\rho_{S}t$ and $F_{C}/\mu^{2}=\left[{\left(\rho_{P}h\right)}^{3/2}/\left(\rho_{F}D^{1/2}\right)\right]f_{c}$.

The same strategy is applied for the prediction of the relation between flutter frequency and the mass ratio as represented in Figure 13. The mass ratio can be defined mathematically as $\mu =\rho_{F}L/\rho_{S}t$ where $F_{C}/\mu^{2}=\left[{\left(\rho_{P}h\right)}^{3/2}/\left(\rho_{F}D^{1/2}\right)\right]f_{c}$ is an ordinate, $F_{C}$ is the reduced critical frequency and $f_{c}$ is the critical frequency. From the experimental and numerical data represented in Figure 13, it is observed that the trend followed by the $F_{C}$ is almost the same as followed by the $U_{RC}$ in Figure 12 for different flag lengths. From Figure 12 and Figure 13 it is observed that flags with smaller lengths are highly sensitive to the critical velocity and frequency respectively while flags with larger lengths are less sensitive to the critical flow velocity and frequency respectively.

## 5. Conclusions

The main aim of this paper was to analyze the performance evaluation of the PZT energy harvester based on the flag-flutter mechanism and flutter instabilities associated with it. The numerical analysis was carried out in MSC Nastran and the experimental campaign was carried out in a subsonic wind-tunnel. The optimal resistance is predicted to be 0.33 MΩ and voltage generated at this particular resistance is 35 V. There is overall no mode shape variation in the flutter mode vibration of numerical and experimental data although the torsion is not predicted numerically. From the hysteresis loop of the bifurcation diagram, it was observed that critical flow velocity for the Al patched flag and PZT patched flag (for both cases i.e., open circuit and optimal resistance) is 23 m/s and 25 m/s respectively. The bifurcation diagram obtained from the experimentation is in a knee shaped sub-critical bifurcation of fifth-order fitting. From flutter boundary conditions, it was observed that the flags with a small length are more sensitive to the flutter velocity as compared to the flags with large lengths. Moreover, the numerical data was found to be in good agreement with the experimental data. The designed PZT energy harvester via flag-flutter mechanism is suitable for energy harvesting in aerospace engineering applications i.e., in high altitude platforms, stratospheric probe balloons, and unmanned aerial vehicles.

## Figures and Tables

**Table 1:** Power generated by various piezoelectric (PZT) aeroelastic energy harvesters (PAEHs) based on flutter mechanism [35].

Type of PZT	PZT Layer(s)	Output Power (mW)
PZT-5A	1	0.003
PSI-5A4E	1	0.2
QP 10N	2	2.2

**Table 2:** Dimensions of flag in numerical campaign (cm).

Material	Length	Width	Thickness
Piezoelectric patch	6.1	3.5	0.01
Fibreglass	15:3:38	6	0.05
Aluminium patch	5	3	0.01

**Table 3:** Material properties.

Properties (unit)	Symbol	Glass Fiber	PIC 255
Young’s Modulus (GPa)	$E_{1}=E_{2}$	21.49	62.1
	$E_{3}$	10	48.3
Shear Modulus (GPa)	$G_{12}$	4	23.5
	$G_{23}=G_{31}$	4	21
Poisson’s Ratio	$\nu_{12}$	0.2	0.32
	$\nu_{23}=\nu_{31}$	0.2	0.44
Density (kg/m${}^{3}$)	ρ	1900	7800

**Table 4:** Nodes and elements for discretization.

Geometry	Nodes	Elements
PZT patch	465	420
Al patch	273	240
PIC 255	273	240
Kapton layers	261	180
Flag (Total)	1765 (17 cm) to 2625 (38 cm)	1344 (17 cm) to 2496 (38 cm)

**Table 5:** Dimensions of flag in experimental campaign (cm); these flags are represented in Figure 4.

	Length	Width	Thickness
Al patched flag	15:3:38	6	0.05
PZT patched flag	29	6	0.05

## Abbreviations

The following abbreviations are used in this manuscript:

*A*	Flag Tip Amplitude
Al	Aluminium
$C_{p}$	Piezoelectric Inherent Capacitance
*d*	Piezoelectric Patch Width
$e_{31}$	Piezoelectric Constant in 31 Coupling Direction
$f_{c}$	Critical Frequency
$F_{C}$	Reduced Critical Frequency
FSI	Fluid-Structure Interaction
*h*	Flag Thickness
$k_{ME}$	Electro-mechanical Coupling Factor
*L*	Length of Flag
LCOs	Limit Cycle Oscillations
PAEH	Piezoelectric Aeroelastic Energy Harvesting
PEH	Piezoelectric Energy Harvesting
PZT	Piezoelectric
*q*	Electrical Charge
*R*	External Resistance
$U_{C}$	Critical Flow Velocity
$U_{RC}$	Reduced Critical Velocity
*V*	Electric Potential
μ	Mass Ratio
$\rho_{F}$	Density of Airflow
$\rho_{S}$	Density of Flag
ξ	Flag Tip Displacement
